# Insights into mRNP biogenesis provided by new genetic interactions among export and transcription factors

**DOI:** 10.1186/1471-2156-13-80

**Published:** 2012-09-10

**Authors:** Francisco Estruch, Christine Hodge, Natalia Gómez-Navarro, Lorena Peiró-Chova, Catherine V Heath, Charles N Cole

**Affiliations:** 1 Departamento de Bioquímica y Biología Molecular, Universitat de Valencia, Burjassot, Spain; 2 Departments of Biochemistry and Genetics, Geisel School of Medicine at Dartmouth, Hanover, New Hampshire, NH, USA

**Keywords:** mRNA export, Transcription, Dbp5p, Mex67p, Nuclear Pore Complex

## Abstract

**Background:**

The various steps of mRNP biogenesis (transcription, processing and export) are interconnected. It has been shown that the transcription machinery plays a pivotal role in mRNP assembly, since several mRNA export factors are recruited during transcription and physically interact with components of the transcription machinery. Although the shuttling DEAD-box protein Dbp5p is concentrated on the cytoplasmic fibrils of the NPC, previous studies demonstrated that it interacts physically and genetically with factors involved in transcription initiation.

**Results:**

We investigated the effect of mutations affecting various components of the transcription initiation apparatus on the phenotypes of mRNA export mutant strains. Our results show that growth and mRNA export defects of *dbp5* and *mex67* mutant strains can be suppressed by mutation of specific transcription initiation components, but suppression was not observed for mutants acting in the very first steps of the pre-initiation complex (PIC) formation.

**Conclusions:**

Our results indicate that mere reduction in the amount of mRNP produced is not sufficient to suppress the defects caused by a defective mRNA export factor. Suppression occurs only with mutants affecting events within a narrow window of the mRNP biogenesis process. We propose that reducing the speed with which transcription converts from initiation and promoter clearance to elongation may have a positive effect on mRNP formation by permitting more effective recruitment of partially-functional mRNP proteins to the nascent mRNP.

## Background

In eukaryotic cells, synthesis of RNA and protein occur in separate cellular compartments, necessitating the continuous transport of macromolecules between the nucleus and cytoplasm. The transport of both RNAs and proteins takes place through the nuclear pore complexes (NPCs) in an energy-dependent process mediated by saturable transport receptors (for reviews, see [1,2]). During mRNA biogenesis, mRNAs are packaged into ribonucleoprotein complexes (messenger ribonucleoprotein particles, mRNPs). Many of the protein components of mRNPs are removed from mRNPs as they are translocate through NPCs. The essential mRNA export factor Dbp5p/Rat8p, a member of the DEAD-box family of proteins [3] binds to the cytoplasmic filaments of the NPC, where its ATPase is activated by Gle1p triggering removal of mRNP proteins [4,5]. There is evidence that the mRNA export receptor Mex67p and the RNA-binding protein Nab2p, both of which accompany the mRNP through NPCs, are removed from mRNPs by Dbp5p acting at the cytoplasmic filaments [6,7]. However, Dbp5p is not restricted to the NPC. It shuttles between nucleus and cytoplasm and can be detected throughout the cell [8,9]. Its homologue in *Chironomus titans* is recruited to mRNA during transcription [10]. Consistent with this, we identified both genetic and physical interactions between Dbp5p and factors involved in transcription initiation, including TFIIH and the C-terminal domain (CTD) of RNA polymerase II (RNA pol II) [11].

The various steps of mRNP biogenesis are coordinated and this is reflected in the extensive interactions that occur between RNA pol II, other components of the transcription machinery, and mRNA processing factors (for review see [12-14]). Several studies have established links between transcription elongation and mRNA export (for review see [15]). The TREX complex includes components involved in transcription elongation (the four subunits of the THO complex) and the essential mRNA export factors Sub2p and Yra1p [16,17]. The interactions between Sub2p, Yra1p and Mex67p lead to a model of sequential binding where Sub2p is recruited by THO and brings Yra1p that works as an adaptor of Mex67p interaction with the transcript [15,18]. Dbp5p also interacts physically with Yra1p in yeast cells and its ortholog, Aly/REF in mammalian cells [19]. Since Yra1p does not shuttle, it appears to be removed from the mRNP prior to its passage through the NPC [6]. We and others have demonstrated that loss-of-function mutations in some components of the basic transcription machinery are able to suppress the growth and mRNA export defects observed in *dbp5* or *sub2* mutant strains [11,20]. One possible explanation for this is that a reduction in the transcription rate could enhance proper formation of mRNPs under condition when some of the proteins needed for mRNP biogenesis are compromised but still functioning [11,21].

To further our understanding of the linkage between nuclear events of mRNA biogenesis and mRNA export, we investigated the effect of mutations affecting various components of the transcription initiation apparatus on the phenotypes of mRNA export mutant strains. Our results show that growth and mRNA export defects of *dbp5* and *mex67* mutant strains can be suppressed by mutation of specific transcription initiation components, but suppression was not observed for mutants acting in the very first steps of the pre-initiation complex (PIC) formation. The data suggest that reducing the speed with which a transcription event converts from initiation and promoter clearance to elongation can have a positive effect on mRNP formation in *dbp5* and *mex67* mutant strains. We hypothesize that this occurs in mutant strains by permitting more effective recruitment to the nascent mRNP of key proteins that are either partially-functional in mutant strains or of limiting abundance.

## Results

### The synthetic lethality of bur6 with dbp5 mutations can be suppressed by overexpression of a truncated allele of RNA pol II subunit RPB2

**Figure 1:**
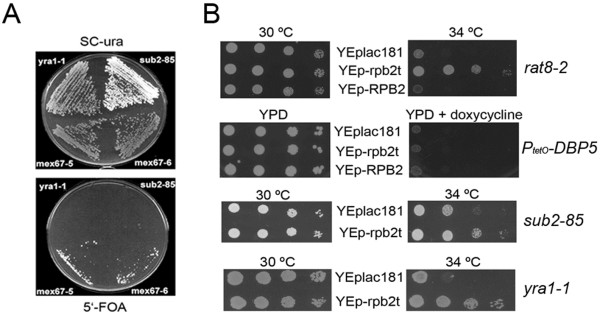
**Genetic interactions among mutants affecting mRNA export factors Dbp5p/Rat8p, Mex67p, Sub2p and Yra1p, and the transcription factor Bur6p and truncated Rpb2tp.** (**A**) Synthetic lethality between *bur6-ts* and *yra1-1* or *sub2-85*. Double mutants *yra1-1 bur6-ts* [pYRA1/URA3] (*yra1-1* in the Figure), *sub2-85 bur6-ts* [pSUB2/URA3] (*sub2-85* in the Figure), *mex67-5 bur6-ts* [pMEX67/URA3] (*mex67-5* in the Figure) and *mex67-6 bur6-ts* [pMEX67/URA3] (*mex67-6* in the Figure) were streaked on SC-ura (as control) or 5^′^-FOA (to analyze synthetic lethality) and incubated at 30 °C for 4 days. (**B**) *rat8-2* mutant was transformed with a C-terminal deletion of *RPB2* carried on the multicopy plasmid YEplac181 (YEp-rpb2t), the wild type *RPB2* gene cloned in YEplac181 (YEp-RPB2) or the empty vector (YEplac181). Serial dilutions (1:10) of transformed cells were spotted onto YPD plates and incubated for 3 days at different temperatures. A yeast strain carrying the *DBP5* gene under the control of the regulatable promoter containing *tet*O (*PtetO-DBP5*) was transformed with the indicated plasmids. Transformed cells were spotted onto YPD or YPD containing 10 mg/L doxycycline and incubated for 3 days at 30 °C. *sub2-85* and *yra1-1* mutants were transformed with the YEp-rpb2t plasmid or the empty vector YEplac181 and growth was analyzed by spotting serial dilutions of the transformants on YPD plates and incubation at different temperatures.

In previous work we identified a variety of genetic interactions between *DBP5* and different factors involved in transcription initiation [11]. Mutations of *dbp5* were synthetically lethal with mutations in the gene encoding Bur6p [11]. Bur6p together with Ydr1p form the transcription repressor NC2 [22]. *In vitro*, the NC2 complex interacts with TBP and blocks the association of TFIIA and TFIIB [22,23]. As can be observed in Figure 1A, *bur6* mutations are also synthetically lethal with *yra1-1* and *sub2-85* but not with *mex67-5* or *mex67-6*, although *mex67 bur6* double mutants are impaired in growth and show a reduced viability (Figure 1A; results not shown).

We attempted to suppress the synthetic lethal phenotype of the double *bur6-1 rat8-2/dbp5* mutant by transforming the double mutant strain carrying the wild-type *DBP5* gene on a *URA3/CEN* plasmid with a high-copy-number YEp13-based library. We selected transformants on SC-leucine plates and replica-plated these onto 5′-FOA containing plates. As expected, plasmids carrying the *BUR6* wild-type gene were isolated (n = 23), but no plasmids containing the *DBP5* wild-type gene were recovered. Based on prior experiments using the same library (our unpublished results), we believe that the library lacks a plasmid containing *DBP5*. Besides plasmids containing *BUR6,* we isolated six independent clones carrying plasmids containing inserts of chromosome XV that included an N-terminal fragment of the *RPB2* gene, the *YOR152c* ORF and an N-terminal fragment of the *PDR5* gene. By subcloning, we mapped the suppressor activity to the truncated *RPB2* gene. This allele encodes the first 379 amino acids (total length of 1,224 residues) and was named *rpb2t*.

The effect of *rpb2t* on the *bur6-1 rat8-2* double mutant prompted us to determine whether expression of *rpb2t* from a high copy plasmid would be able to suppress the growth defect shown by individual *dbp5* and *bur6* mutants. The effect of Rpb2t on the growth defect caused by the depletion of Bur6p has been previously reported [24]. Figure 1B shows that Rpb2tp, but not wild-type Rpb2p, partially suppresses the growth defect of *rat8-2* at 34°C. However, when the YEp-rpb2t plasmid was introduced into a yeast strain where the *DBP5* gene was expressed under the control of the doxycycline-repressible *tetO* promoter, we did not observe any growth improvement in the presence of doxycycline (Figure 1B). Therefore, *rpb2t* is not a bypass suppressor, since expression of Rpb2tp does not suppress the growth defect caused by depletion of Dbp5p. We also examined the effect of *rpb2t* on other mutants affecting important mRNA export factors. Figure 1B shows that *rpb2t* improved the growth of the *sub2-85* and *yra1-1* mutants at a semi-restrictive temperature. However, it had no effect on the growth of the *mex67-5* or *mex67-6* strains (data not shown). Suppression by *rpb2t* thus correlates with synthetic lethality with *bur6-1* in that *rat8-2, yra1-1* and *sub2-85* all show synthetic lethality with *bur6-1* but *mex67-5* and *mex67-6* do not.

### A reduction of the amount of RNA pol II does not suppress the growth defect of the rat8-2 mutant

**Figure 2:**
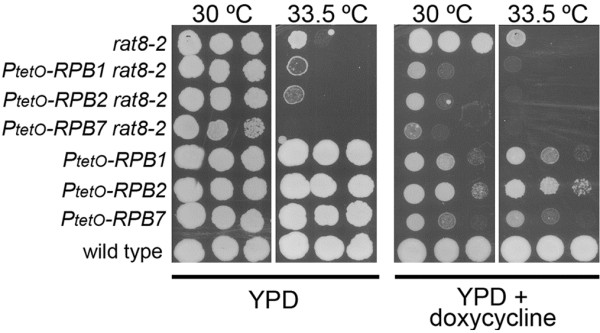
**Depletion of RNA pol II subunits does not suppress the growth defects of the*rat8-2*mutant.** Wild type, single and double mutants were spotted in YPD or YPD containing 1 mg/L doxycycline and incubated at the indicated temperatures for 4 days.

We considered the possibility that truncated Rpb2tp might cause a reduction in RNA pol II activity, as a result of the presence of non-functional RNA pol II holoenzymes (those containing the truncated Rpb2tp) competing with functional ones. To check if a reduction of the amount of different subunits of RNA pol II would be able to suppress the growth defect shown by the *rat8-2* mutant at restrictive temperature, we tested strains in which the promoters of different RNA pol II subunits had been replaced by the regulatable *tetO* promoter. Figure 2 shows that strains expressing the *RPB1*, *RPB2* or *RPB7* genes under the control of the *tetO* promoter were able to grow to different extents at low doxycycline concentration (1 μg/ml). However the reduction of *RPB1*, *RPB2* and *RPB7* expression further impaired, rather than enhanced, the growth defect of the *rat8-2* mutant at the semi-restrictive temperature. In addition, we observed that deletion of the non-essential *RPB9* gene also had a negative effect on the growth of *rat8-2* at 34°C (data not shown). Therefore, these findings demonstrate that reducing the amount of RNA pol II is not sufficient to suppress the growth defect of *rat8-2*.

### Genetic interactions of mRNA export factor mutants with components of RNA pol II general transcription factors (GTFs)

**Figure 3:**
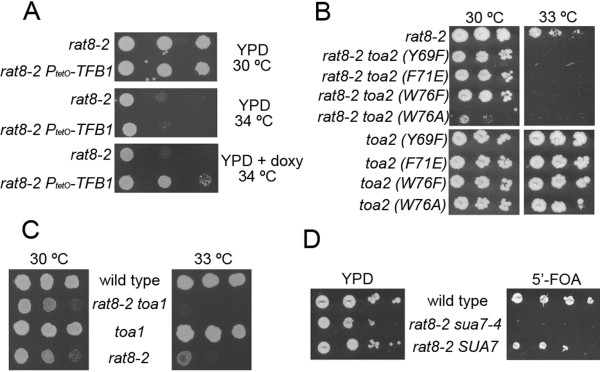
**Genetic interactions between mRNA export factors and components of the transcription machinery.** (**A**) Suppression of *rat8-2* by depletion of the TFIIH component Tfb1p. *rat8-2* single mutant and *rat8-2 P.*_*tetO*_*-TFB1* double mutant were spotted onto YPD or YPD containing 10 mg/L of doxycycline plates and incubated at the indicated temperatures for 4 days. (**B**) Synthetic sickness of *rat8-2* and *toa2* mutations. Serial dilutions of single and double mutants strains were spotted onto YPD plates and incubated at different temperatures for 4 days. (**C**) Mutation in *toa1* impairs growth of *rat8-2* mutants. Strains analyzed were segregants from the cross CSY550 (*rat8-2*) x MY1870 (*toa1-2*). Cells were spotted onto a YPD plate and incubated for 3 days at the indicated temperatures. (**D**) Synthetic lethality between *rat8-2* and *sua7-4*. Double mutants *rat8-2 sua7:: LEU2* [p*SUA7/URA3*] carrying the wild type *SUA7* gene or the mutant allele *sua7-4* in a *HIS3/CEN* plasmid were spotted on YPD or 5′FOA plates and incubated at 30 °C for 4 days

The suppression of *dbp5/rat8* mutations by mutations in *SSL1*, encoding a subunit of TFIIH [11] prompted us to analyze the genetic interactions of *rat8-2* with other general transcription factors (GTFs). We replaced the promoter of the TFIIH-component *TFB1* with the doxycycline-repressible promoter containing *tetO*. As shown in Figure 3A, the partial depletion of Tfb1p alleviated the growth defect of the *rat8-2* mutant at 34°C.

Next, we analyzed the genetic interactions between *rat8-2* and mutants affecting TFIIA and TFIIB. We tested the TFIIA components encoded by the *TOA1* and *TOA2* genes. In the case of *TOA1*, we combined the *rat8-2* mutation with *toa1-2*, an allele isolated as suppressor of a defect in NC2 function [25]. As it has been mentioned above, mutations of *dbp5* were found to be synthetically lethal with mutations in the gene encoding the NC2-component Bur6p. In the case of Toa2p, we used four different single point mutants of *TOA2*[26]. Both *rat8-2 toa2* (Figure 3B) and *rat8-2 toa1* (Figure 3C) double mutants grew notably more slowly at semi-restrictive temperature than the single mutants. Similarly, we observed that *rat8-2* was synthetically lethal at 30°C with a temperature sensitive allele of *SUA7* (*sua7-4*)*,* encoding TFIIB [27], although both single mutants grew well at 30° (Figure 3D and data not shown).

Taken together, these genetic analyses indicate that the pattern of synthetic lethality and suppression cannot be explained by the simple model related to increasing or decreasing the overall amount of transcription.

### Genetic interaction between ssl1 and mex67 mutants

**Figure 4:**
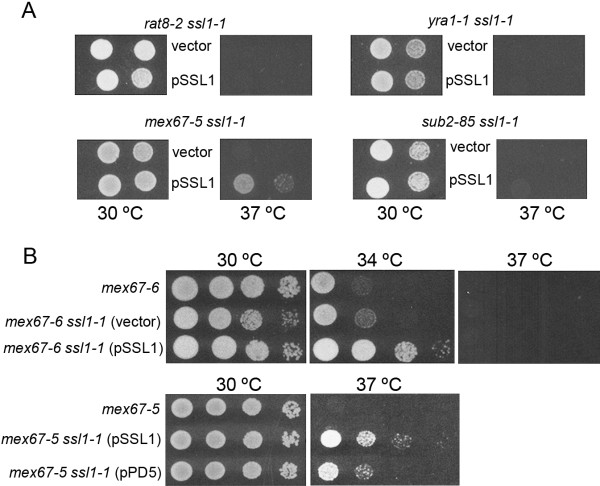
**Suppression of*mex67*ts growth defect by*ssl1-1.*** (**A**) *rat8-2*, *yra1-1*, *mex67-5* and *sub2-85* mutations were combined with *ssl1-1*. Double mutant strains were transformed with a CEN plasmid containing the *SSL1* wild type gene (pSSL1), or the empty vector pRS316 (vector). Serial dilutions (1:10) were spotted onto SC-ura plates and incubated for 3 days at different temperatures. (**B**) Suppression of *mex67-6* ts growth defect by *ssl1-1*. Serial dilutions (1:10) of *mex67-6* single mutant and *mex67-6 ssl1-1* double mutant (either transformed with pSSL1 or the empty vector pRS316) were spotted onto YPD plates and incubated for 3 days at different temperatures. Effect of the copy number of the *SSL1* wild type gene on growth of the *mex67-5 ssl1-1* double mutant strain. Serial dilutions (1:10) of *mex67-5* single mutant and *mex67-5 ssl1-1* double mutant transformed with *SSL1* in a CEN/URA3 plasmid (pSSL1) or in a multicopy vector (pPD5) were spotted onto YPD plates and incubated for 3 days at different temperatures.

Mutations in *SSL1* partially suppress the growth defect of *rat8-2* at semi-permissive temperatures but not at the restrictive temperature of 37°C (Figure 4A; [11]). We combined the thermosensitive *ssl1-1* allele with *yra1-1*, *sub2-85* and *mex67-5* and analyzed the growth of the double mutants at the restrictive temperature of 37°C. As can be observed in Figure 4A, the combination of the wild type *SSL1* and the *ssl1-1* mutant allele partially suppressed the growth defect of the *mex67-5* mutant at 37°C although suppression was not observed if only *ssl1-1* was present, presumably because *ssl1-1* cells cannot grow at 37°C [28] and *mex67-5* cannot suppress its growth defect. A similar result was found for *mex67-6*, which has a stronger growth defect than *mex67-5* (Figure 4B). In this case, the double mutant *mex67-6 ssl1-1* grew slightly better than the single *mex67-6* mutant at 34°C. The presence of wild type *SSL1* in addition to *ssl1-1* improved growth of *mex67-6* at 34°C, but cells were still unable to grow at 37°C (Figure 4B).

The unexpected effect of having the simultaneous presence of both wild-type and mutant Ssl1p prompted us to examine how suppression was affected by altering the relative copy numbers of wild type and mutant alleles of *SSL1.* We transformed the *mex67-5 ssl1-1* double mutant strain with a *CEN* (pSSL1) or 2 μm plasmid (pPD5) carrying *SSL1*. Growth of these transformants at different temperatures is shown in Figure 4B. As can be observed, the wild-type *SSL1* gene was required for suppression and suppression was stronger when cells contained fewer copies of the wild type *SSL1* gene (compare growth with pSSL1 and pPD5).

### Mutation of SSL1 suppresses other phenotypes of the mex67-5 mutant

**Figure 5:**
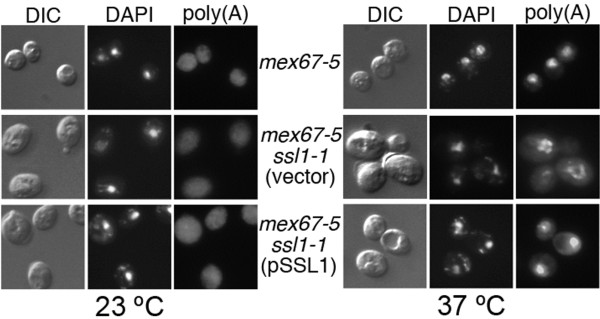
***ssl1-1*mutation affects nuclear accumulation of poly(A)**^**+**^**RNA in*mex67-5*mutant cells.** Single *mex67-5* and double *mex67-5 ssl1-1* [either transformed with the empty vector (vector) or SSL1 in a CEN plasmid (pSSL1)] mutant cells were grown to mid-log phase at 23 °C and incubated for 1 hour at 37 °C. In all the cases, cells were fixed and *in situ* hybridization was performed using a digoxigenin-conjugated oligo(dT) probe, followed by incubation with a FITC-conjugated anti-digoxigenin antibody [poly(A)]. DNA was visualized by DAPI staining.

We previously observed that, at semi-permissive temperature of 34°C, *rat8-2 ssl1-1* double mutant cells accumulated significantly less poly(A)^+^ mRNA in nuclei than did the single *rat8-2* mutant [11]. Since *mex67* mutants have a strong defect in export of poly(A)^+^ RNA at elevated temperatures [29] we examined how the *ssl1-1* mutation would affect the mRNA export defect of the *mex67* mutants. Figure 5 shows that *ssl1-1* altered the distribution of poly(A)^+^ mRNA in *mex67-5* cells. More poly(A)^+^ RNA was cytoplasmic in the *ssl1-1 mex67-5* double mutant than in *mex67-5* cells. In addition, although mRNA still accumulated in the nucleus, it was mainly distributed around the nuclear periphery in double-mutant cells while poly(A)^+^ RNA filled the nucleus in *mex67-5* cells. When the *ssl1-1 mex67-5* double mutant was transformed with the wild type *SSL1* gene on a centromeric plasmid, there was less suppression of the *mex67-5* mRNA export defect, although these cells also showed a stronger cytoplasmic signal for poly(A)^+^ RNA than did *mex67-5* cells. In addition, introduction of *SSL1* eliminated the perinuclear distribution and poly(A)^+^ mRNA could be seen throughout the nucleus (Figure 5). The fact that *SSL1*^*+*^ is required for growth of *mex67-5 ssl1-1* but reduces suppression of the *mex67-5* mRNA export defect by *ssl1-1* suggests that mutant Ssl1-1p protein is responsible for reducing the export defect of *mex67-5* but wild type Ssl1p is required for provision of some Ssl1p function(s) compromised by the *ssl1-1* mutation (see discussion below) .

**Figure 6:**
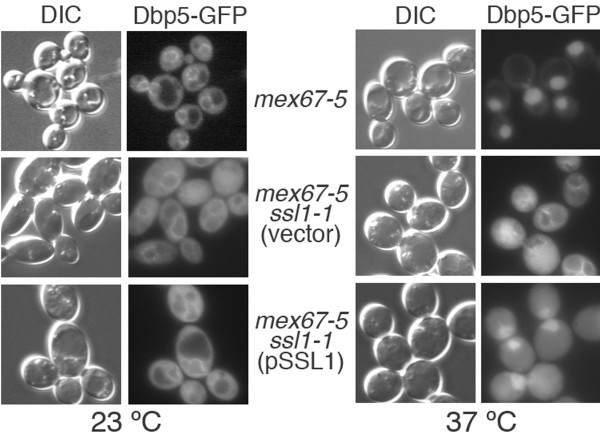
***ssl1-1*mutation affects cellular distribution of Dbp5-GFP in*mex67-5*mutant cells.** Single *mex67-5* and double *mex67-5 ssl1-1* [either transformed with the empty vector (vector) or *SSL1* in a CEN plasmid (pSSL1)] mutant cells were transformed with plasmid pCS835 (expressing a Dbp5-GFP fusion). Cells were grown to mid-log phase at 23 °C and incubated for 10 minutes at at 37 °C.

Previous work has also shown that Dbp5p accumulates in nuclei in several strains with defects in nuclear transport, including *mex67-5*[3]. We found that the nuclear accumulation of Dbp5-GFP observed in the *mex67-5* mutant at 37°C was prevented completely by the *ssl1-1* mutation (Figure 6). However, when the double-mutant *mex67-5 ssl1-1* strain was transformed with wild-type *SSL1* on a centromeric plasmid, we observed an intermediate effect: there was less of an increase in cytoplasmic Dbp5-GFP (although cytoplasmic staining can also be observed at 23°C) and more nuclear Dbp5-GFP than when wild-type *SSL1* was not present (Figure 6).

**Figure 7:**
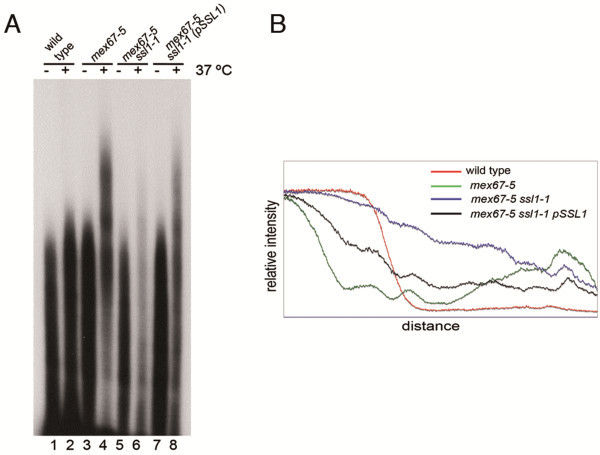
***ssl1-1*mutation affects hyperpolyadenylation in*mex67-5*mutant cells.** (**A**) Poly(A) tail length was determined by isolating total RNA from each strain that had been grown continuously at 23 °C (-) or shifted to 37 °C for 30 min (+). The resulting RNAs were digested to completion with RNases T_1_ and A. The remaining oligo(A) and poly(A) fragments were then end labeled with [^32^P]pCp and RNA ligase and resolved on 9% polyacrylamide-7 M urea-TBE gels Gels were dried and exposed to X-Omat Blue film. (**B**) Densitometric analysis of the poly(A) tail length. Lanes corresponding to the samples obtained at restrictive temperature (37 °C) were densitometered using the Quantity One program (Bio-Rad).

It has been reported that mutations in *MEX67* and other genes encoding proteins involved in mRNA export, including *NUP159*, *GLE1* and *DBP5*, produce nascent transcripts carrying poly(A)^+^ tails roughly 30 residues longer than those observed in wild type cells [30]. We analyzed the effect of the *ssl1-1* mutation on the length of poly(A)^+^ tails produced in *mex67-5* cells (Figure 7A). At restrictive temperature, the amount of poly(A)^+^ RNA produced was reduced in the *mex67-5 ssl1-1* mutant strain (Figure 7A, lane 6) compared to *mex67-5* (lane 4) or wild-type cells (lane 2), consistent with the importance of Ssl1p and TFIIH for transcription. However, although some mRNAs with extended poly(A)^+^ tails were still produced in the *mex67-5 ssl1-1* double mutant, the distribution of poly(A)^+^ tail lengths was shifted towards a wild-type distribution (Figure 7B). When the *mex67-5 ssl1-1* double mutant strain was transformed with the wild type *SSL1* gene, a wild-type level of transcripts was recovered at restrictive temperature (Figure 7A, lane 8), but there was considerably less of a shift towards poly(A)^+^ tails of normal length than when wild-type *SSL1* was not present (Figure 7B).

## Discussion

### Genetic interactions among transcription and export factors suggest complex relationships between transcription and export activities

Previous studies showed that mutations in *SSL1* and *RAD3*, encoding TFIIH components, and *CEG1*, encoding one of the enzymes of the capping apparatus, result in suppression of the growth and mRNA export defects observed in some mRNA export factor mutant strains ([11,20,31], see above). In this report we describe additional genetic interactions between mutations affecting components of the basal transcription machinery and *rat8-2*, *sub2-85*, *yra1-1*, *mex67-5* and *mex67-6*. In addition, mutations in *DBP5*, *YRA1* and *SUB2*, and *MEX67*, are synthetically lethal (or synthetically sick) with mutations in the gene encoding the TBP-binding protein Bur6p (Figure 1A). We also report the suppression of *rat8-2* mutation by partial depletion of Tfb1p (a TFIIH component) and by expression of a truncated form of Rpb2p (referred as Rpb2tp), which is also able to suppress the growth defects of the *yra1-1* and *sub2-85* mutants (Figure 1B).

We and others have suggested that mutations affecting components of the basal transcription machinery suppress defects in mRNA export factors by reducing the rate of formation of mRNP complexes [11,20]. A key question is whether simply reducing the overall rate of mRNP production is sufficient to suppress these defects. The finding that loss-of-function mutations affecting some components of the basic transcription apparatus enhance the growth defect of mRNA export mutants (Figure 3) suggests that mere reduction in the production of mRNP is not sufficient for suppression. The list of mutations without suppressive effect on *rat8-2* includes a mutant allele of *SPT6* (encoding a nucleosome remodeling protein that functions in transcription elongation), a disruption of *ELP3* (encoding a subunit of the elongator complex) or *HPR1* (encoding a subunit of THO/TREX) [11] and alleles affecting the TFIIA (Toa1p and Toa2p) and TFIIB (Sua7p) components. Elp3p, Spt6p, and Hpr1p have been reported to be involved in transcriptional elongation [32-34]. TFIIA and TFIIB are general transcription factors recruited early during preinitiation complex formation. On the other hand, suppression of *rat8-2* has been observed with mutations in two different subunits of the TFIIH complex (Ssl1p and Tfb1p), the capping enzyme Ceg1p and by expression of the truncated Rpb2tp. TFIIH plays a critical role in the formation of the open complex during transcription initiation and in promoter escape, and it has been proposed that the capping enzyme executes a checkpoint during early transcription that assesses whether capping of the nascent transcript has occurred within a specific time window, and if so, allowing elongation to proceed [35-37]. Thus, the pattern of genetic interactions (both suppressive and not suppressive) we have described suggests that suppression occurs with mutants affecting events after formation of the pre-initiation complex but prior to the onset of extensive transcription elongation. These results may indicate that only a limited time window exists for co-transcriptional loading of mRNA export factors onto the nascent transcript. By this scenario, truncated Rpb2tp might act by sequestering some component(s) of the transcription apparatus, resulting in reduced efficiency of some early step of transcription, thereby permitting the partially-defective mutant mRNA export factor to perform its function sufficiently well to support growth.

### Different phenotypes of the mex67 mutant strain can be suppressed by the combination of wild type and mutant SSL1 alleles

In a previous report we showed that some defects of the *rat8-2* mutant could be suppressed by mutations in general transcription factors such as the TFIIH component *SSL1*[11]. Similarly, Jensen et al. [38] found that mutations in *RAD3*, also encoding a TFIIH component, suppressed both growth and mRNA export defects of the *sub2-201* mutant strain. In the studies reported here, we analyzed the effect of the *ssl1-1* mutation on different export factor mutant strains. Our results show that, as occurs with *DBP5*, *ssl1-1* partially suppresses the growth defect of the *mex67-6* mutant at the semi-restrictive temperature of 34°C (Figure 4B). The *mex67-5* mutant strain is able to grow at 34°C but not at 37°C. However, this restrictive temperature could not be used to check the suppression of *mex67-5* by *ssl1-1*, since the *ssl1-1* mutation prevents growth at 37°C [28]. Unexpectedly, we observed partial suppression of the *mex67-5* growth defect at 37°C when we simultaneously introduced the recessive *ssl1-1* mutant allele and the wild-type *SSL1* gene on a centromeric plasmid (Figure 4B). The suppression caused by the *ssl1-1/SSL1* combination was also observed for the *mex67-6* mutant strain, and is sensitive to the ratio of wild type and mutant Ssl1p (Figure 4B).

As occurs with *rat8-2* mutants [11], the *ssl1-1* mutation alters the distribution of poly(A)^+^ mRNA in *mex67-5* cells (Figure 5). Interestingly, suppression of the mRNA export and hyper-polyadenylation defects of *mex67-5* by *ssl1-1* was reduced if wild-type *SSL1* was also present. Our data suggest that wild-type Ssl1p is needed because the Ssl1-1p protein cannot perform all of Ssl1p’s functions at 37°C, although the suppression of *mex67-5* mutant phenotypes by Ssl1-1p indicates that it is performing some functions. The finding that the presence of wild type Ssl1p reduces the degree of suppression of the mRNA export and hyper-polyadenylation defects of *mex67-5* by Ssl1-1p suggests that there may be important difference in mRNPs produced through the action of TFIIH that contains mutant as opposed to wild-type Ssl1p. Perhaps key events related to recruitment of important export factors occur during the period that Ssl1p/TFIIH functions and only those mediated by TFIIH containing mutant Ssl1-1p occur in a manner that permits a later effective recruitment of Mex67-5p. We propose that suppression of the hyper-polyadenylation defect by Ssl1-1p reflects the fact that those mRNPs produced through the action of Ssl1-1p-containing TFIIH undergo more accurate 3′ processing than those produced in *mex67-5* cells (where wild-type Ssl1p is present), thereby reducing hyper-polyadenylation and increasing mRNA export. However, mRNA still accumulates in nuclei in *mex67-5*/*ssl1-1* cells (although accumulation is lower than in *mex67-5* cells). Furthermore, in the presence of *ssl1-1* but not *SSL1,* the distribution of the poly(A)^+^ signal inside the nucleus differs from that observed in the *mex67-5* single mutant, with poly(A)^+^ mRNA accumulating primarily at the nuclear periphery in *mex67-5/ssl1-1* cells (Figure 5). The accumulation of poly(A)^+^ RNA in nuclear foci observed in the *mex67-5* mutant is thought to reflect retention of mRNA near sites of transcription, whereas the accumulation at the nuclear periphery observed in the *mex67-5 ssl1-1* strain suggests that the *ssl1-1* mutation permitted release of poly(A)^+^ mRNP from intranuclear sites, possibly reflecting an improvement in the quality of the mRNP produced. The fact that a partial mRNA export block remains in *mex67-5/ssl1-1* cells could reflect the fact that the presence of *ssl1-1* does not return the accuracy of mRNP production to wild-type levels and that some mRNPs are not in an exportable configuration. Alternatively, it may be that the Mex67-*5*p protein is less functional than wild type Mex67p for functions that occur during translocation through the NPC. Since *mex67-5/ssl1-1* cells grow, there must be an increase in mRNA export. This is more easily explained by improved quality of the mRNP due to Ssl1-1p than to Ssl1-1p acting to enhance Mex67-5p functionality during NPC translocation, since Ssl1p is not part of the exported mRNA and is not expected to have any direct affect on the actual mRNA export event. It is interesting that a modest increase in the amount of cytoplasmic poly(A)^+^ RNA allows *mex67-5/ssl1-1* cells to grow whereas *mex67-5* cells do not. At present, it is not technically possible to determine if there are important qualitative changes in the organization or composition of mRNPs when the *ssl1-1* allele is present. In a large number of experiments over many years and in different mutants we have observed that cellular growth does not require levels of cytoplasmic poly(A)^+^ RNA equal to or even near what is observed in wild-type cells. Often cells with substantial nuclear accumulation of poly(A)^+^ RNA and a modest cytoplasmic signal are able to grow, albeit at a reduced rate in many cases. This is clearly the case with the *nup159ΔN* mutant which grows at a moderate rate at 25°C despite strong accumulation of poly(A)^+^ RNA in nuclei [3].

Nuclear accumulation of Dbp5-GFP also occurs in *mex67-5* cells [3]. This accumulation is not observed in the double *mex67-5 ssl1-1* mutant, whereas in the *mex67-5 ssl1-1/SSL1* strain, there is a strong nuclear signal, although accompanied by an increased cytoplasmic staining, relative to that seen in *mex67-5* cells (Figure 6). Although the causes for the nuclear accumulation of Dbp5p in *mex67* remain to be clarified, our results suggest that this phenotype could be related to mRNP retention at transcription site foci due to the *mex67-5* mutation. These mRNPs would contain Dbp5-GFP resulting in accumulation of Dbp5-GFP in nuclei. The nuclear accumulation of Dbp5p could also contribute to the hyper-polyadenylation defect since this may lead to reduced levels of Dbp5p associated with NPC filaments, where it functions to mediate remodeling of the mRNP complex. In this situation, removal of shuttling mRNA binding proteins by Dbp5p might be less efficient and the mRNPs produced might have a defective composition due to sub-optimal levels of shuttling mRNA-binding proteins that become part of mRNPs in the nucleus. Additional work will be required to elucidate how Dbp5p participates in the assembly of an export competent mRNP and what distinguishes export-competent mRNPs from those that accumulate in nuclei.

## Conclusions

Our previous results and those reported here suggest roles for Dbp5p both during transcription and at the NPC during mRNP translocation. The finding that mutations in components of TFIIA and TFIIB or a reduction in the amount of RNA pol II exacerbate the growth defect of *dbp5/rat8-2* at semi-restrictive temperature indicates that mere reduction in the amount of mRNP produced is not sufficient to suppress the defects caused by a defective Dbp5p. The data suggest that suppression occurs only with mutants affecting events within a narrow window of the mRNP biogenesis process. We propose that reducing the speed with which transcription converts from initiation and promoter clearance to elongation may have a positive effect on mRNP formation in *dbp5* mutant strains by permitting more effective recruitment of mRNP proteins to the nascent mRNP.

## Methods

### Yeast strains and genetic methods

Yeast strains used in this study are listed in Table 1. Strains were grown using standard methods. For growth assays, yeast cells were diluted to the same OD_600_, and serial dilutions (1:10) were spotted onto YPD or selective plates and incubated at various temperatures. 5′ fluoro-orotic acid (5′-FOA) was added to synthetic complete media at 1 g/L. Doxycycline was added to YPD plates at 1 or 10 mg/L.

**Table 1:** Yeast strains used in this study

**Strain**	**Genotype**	**Source**
FY23	*MATa leu2Δ1 ura3-52 trp1Δ63*	[39]
FY86	*MATα leu2Δ1 ura3-52 his3Δ200*	[39]
CSY550	*MATa leu2Δ1 ura3-52 trp1Δ63 rat8-2*	[8]
PDY4	*MATa ura3-52 his3Δ200 ssl1-1 rat8-2*	[11]
CSY564	*MATα leu2Δ1 ura3-52 his3Δ200 rat8-2* [pRAT8.31]	[11]
GY215	*MATa ura3-52 trp1Δ63 his4-912δ lys2-128δ bur6-1*	G. Prelich
PDY6	*MATα leu2Δ1 ura3-52 lys2-128δ rat8-2 bur6-1* [pRAT8.31]	[11]
CSY560	*MATa leu2Δ1 ura3-52 trp1Δ63 rat8-2* [pRAT8.31]	[11]
CS41-4.3	*MAT? ura3-52 leu2-3 his3-11 trp1-1 ade2-1 bur6:: HIS3* [p*bur6-ts/CEN/LEU2*]	D. Reinberg
YMH202	*MAT? ura3-52 leu2-3 his3-11 trp1-1 ade2-1 bur6:: HIS3* [p*BUR6/CEN/URA3*]	D. Reinberg
*mex67-5*	*MATa ade2 leu2 ura3 his3 trp1 mex67::HIS3* [p*mex67-5*/*TRP1/CEN*]	[29]
*yra1-1*	*MATa ade2 leu2 ura3 his3 trp1 yra1::HIS3* [p*yra1-1*/*TRP1/CEN*]	[18]
*sub2-85*	*MATa leu2 ura3 his3 trp1 sub2::kanMX4* [p*sub2-85*/*TRP1/CEN*]	[40]
*yra1-1 bur6-ts*	*MAT? ade2-1 trp1 ura3 his3 leu2 bur6:: HIS3 yra1::HIS3* [p*bur6-ts/CEN/LEU2*] [p*yra1-1*/*TRP1/CEN*] *[pYRA1/URA3/CEN*]	This study
*sub2-85 bur6-ts*	*MAT? trp1 ura3 his3 leu2 bur6::HIS3 sub2::kanMX4* [p*bur6-ts/CEN/LEU2*] [p*sub2-85*/*TRP1/CEN*] *[*p*SUB2/URA3/CEN*]	This study
*mex67-5 bur6-ts*	*MAT? ade2-1 trp1 ura3 his3 leu2 bur6::HIS3 mex67::HIS3* [p*bur6-ts/CEN/LEU2*] [p*mex67-5*/*TRP1/CEN*] *[*p*MEX67 URA3/CEN*]	This study
*mex67-6 bur6-ts*	*MAT? ade2-1 trp1 ura3 his3 leu2 bur6::HIS3 mex67::HIS3* [p*bur6-ts/CEN/LEU2*] [p*mex67-6*/*TRP1/CEN*] [p*MEX67 URA3/CEN*]	This study
*mex67-5 ssl1-1*	*MAT? ade2 trp1? leu2 ura3 ssl1-1 mex67::HIS3* [p*mex67-5*/*TRP1/CEN*]	This study
*mex67-6 ssl1-1*	*MAT? ade2 trp1? leu2 ura3 ssl1-1 mex67::HIS3* [p*mex67-6*/*TRP1/CEN*]	This study
*sub2-85 ssl1-1*	*MAT? his3 ura3 sub2::kanMX4* [p*sub2-85*/*TRP1/CEN*]	This study
*TOA1 rat8-2*	*MAT? leu2 his3 ura3 rat8-2*	This study
*toa1-2 rat8-2*	*MAT?leu2 trp1 rat8-2 toa1-2*	This study
*toa1-2 DBP5*	*MAT? leu2 toa1-2*	This study
*TOA1 DBP5*	*MAT? leu2 his3*	This study
MY1870	*MATα trp1 ura3-52 leu2::PET56 gal2 gcn4D toa1-2*	[25]
*toa2 (Y68F)*	*MATα toa2::HIS3 ade1 ura3-52 leu2Δ1* [p*toa2(Y68F)/LEU2/CEN*]	[26]
*toa2 (F71E)*	*MATα toa2::HIS3 ade1 ura3-52 leu2Δ1* [p*toa2(F71E)/LEU2/CEN*]	[26]
*toa2 (W76F)*	*MATα toa2::HIS3 ade1 ura3-52 leu2Δ1* [p*toa2(W76F)/LEU2/CEN*]	[26]
*toa2 (W76A)*	*MATα toa2::HIS3 ade1 ura3-52 leu2Δ1* [p*toa2(W76A)/LEU2/CEN*]	[26]
*rat8-2 toa2 (Y68F)*	*MAT? toa2::HIS3 rat8-2 ura3-52 leu2Δ1 trp1Δ63* [p*toa2(Y68F)/LEU2/CEN*]	This study
*rat8-2 toa2 (F71E)*	*MAT? toa2::HIS3 rat8-2 ura3-52 leu2Δ1 trp1Δ63* [p*toa2(F71E)/LEU2/CEN*]	This study
*rat8-2 toa2 (W76F)*	*MAT? toa2::HIS3 rat8-2 ura3-52 leu2Δ1 trp1Δ63* [p*toa2(W76F)/LEU2/CEN*]	This study
*rat8-2 toa2 (W76A*)	*MAT? toa2::HIS3 rat8-2 ura3-52 leu2Δ1 trp1Δ63* [p*toa2(W76A)/LEU2/CEN*]	This study

### High copy suppression screen

Multicopy suppressors of the synthetic lethality between *bur6-1* and *rat8-2* mutant alleles were obtained by transforming the *bur6-1 rat8-2* double mutant strain carrying the wild type *DBP5* gene in a *URA3/CEN* plasmid (pCS831) with a yeast genomic library cloned in the YEp13 vector (*LEU2*/2 μm). Transformants (40,000) were grown on selective medium at 30°C for 3 days and then replica plated onto 5^′^-FOA-containing plates and incubated two additional days at 30°C. Plasmids able to suppress the synthetic lethality were isolated and used to re-transform the *bur6-1 rat8-2* double mutant. For the positive clones, insert ends were determined by DNA sequencing, revealing that six of them were identical. In these plasmids, the suppressor activity was mapped to a *Xba*I fragment (one of the *Xba*I sites in the YEp13 vector and the second in the genomic insert) that included sequences corresponding to the promoter and 5′-terminal part of the *RPB2* gene. The *Xba*l fragment was subcloned into the YEplac181 yielding the YEp-rpb2t plasmid.

### Plasmids

The plasmid overexpressing wild type *RPB2 gene* has been described previously [24]. Plasmid pPD5 (containing the *SSL1* gene in a multicopy vector) has been described previously [11]. To clone the *SSL1* gene in a centromeric plasmid, an *Eag*I-*Bam*HI fragment from pPD5 was inserted between the *Eag*l and *Bam*HI sites of the pRS314 or pRS316 vectors [41]. Plasmid pCS835 expressing a Dbp5-GFP fusion has been described [3].

### *In situ* hybridization and microscopy

To localize poly(A)^+^ RNA within cells, we used *in situ* hybridization with an oligo(dT)_50_ probe coupled to digoxigenin, which was performed as described previously [42]. Fluorescence images were examined and photographed using an Axiophot microscope (Zeiss) and a CCD camera.

### poly(A) tail length measurements

poly(A)^+^ tail length measurements was performed as described by [43]. Briefly, 10 μg of total RNA was digested with 2 μg of bovine pancreatic RNase per ml and 1,000 U of RNase T1 per ml to produce a RNA population consisting only of poly(A) tails. Each RNA sample was then end labeled with 100 μCi of [5^′^^32^P]cytidine 3^′^,5^′^ bis(phosphate) (NEN Life Science Products, Boston, Mass.) in a solution containing 50 mM Tris–HCl (pH 7.9), 15 mM MgCl_2_, 3.3 mM dithiothreitol (DTT), 2% (vol/vol) dimethyl sulfoxide, 10 mg of bovine serum albumin per ml, 25 μM ATP, and 10 U of T4 RNA ligase for 21 h at 4°C. RNA samples were then extracted with phenol and precipitated with ethanol. Pellets were then resuspended in a solution containing 96% formamide, 0.1% bromophenol blue, and 0.1% xylene cyanol, denatured by boiling, and resolved on a 10% polyacrylamide-1 M urea-TBE (Tris-borate-EDTA) gel. Gels were dried and exposed to X-Omat Blue film (Kodak, Rochester, N.Y.).

## Abbreviations

NPC: Nuclear pore complex; CTD: Carboxi-terminal domain; PIC: Pre-initiation complex; mRNP: Messenger ribonucleoprotein particle.

## Competing interests

The authors declare that they have no competing interests.

## Authors’ contributions

FE carried out most of the experiments reported in the manuscript, participated in the design of the experiments, and wrote the paper. CH, NG-N, LP-C and CVH carried out some experiments. CNC conceived the study and wrote the paper. All authors read and approved the final manuscript.
